# Supplementary material for: Content-rich biological network constructed by mining PubMed abstracts
Source: BMC Bioinformatics. 2004 Oct 8;5:147. doi: 10.1186/1471-2105-5-147 (PMC528731; doi:10.1186/1471-2105-5-147)
Supplement: Additional File 2 — The original results of the above study (non-essential files are deleted to keep the file size under the limit set by BMC bioinformatics). [file 1471-2105-5-147-S2.bz2 › chilibotAdditionalFile2/dip05/486ID9312096E10581/html/BLR1_CCL5.html]

 


 **BLR1** and **CCL5** 
  
Found 5 abstracts in PubMed, retrieved 05.  
 

 What does Google say? 
 PDF only 
| .edu only 

---

**Interactive relationship** (e.g. stimulation, inhibition, etc)

**Neutral relationship**- However, none of a series of radiolabelled chemokines IL 8, NAP 2, GRO alpha, PF4, IP10, MCP 1, MCP 2, MCP 3, I 309, RANTES  [ **CCL5** ]  and MIP 1 alpha and other ligands C3a and leukotriene B4 bound to Jurkat transfectants that stably expressed either MDR15  [ **BLR1** ]  or  **BLR1**  mRNA.  Ref: 7639692 Biochem J, 1995

**Non-interactive relationship** (e.g. studied together, co-existance, homology, etc.)

- Other changes observed, including alteration in the gene expression profile of adhesion molecules and chemokine receptors such as CD44H, CD 49B, Rantes  [ **CCL5** ]  R, CXCR5  [ **BLR1** ]  and CD37, suggest differences in trafficking potential between the populations studied.  Ref: 11529869 Br J Haematol, 2001
- Although several of the analyzed receptors including CCR6, CCR7, CXCR4, and CXCR5  [ **BLR1** ]  were not differentially expressed on gammadelta vs alphabeta T cells, gammadelta T cells expressed strongly increased levels of the RANTES  [ **CCL5** ]  macrophage inflammatory protein 1alpha 1beta receptor CCR5 and also enhanced levels of CCR1 3 and CXCR1 3.  Ref: 11994442 J Immunol, 2002
